# Role of mixed reality in the workflow of complex neurooncological surgeries: a case analysis in thalamic surgery

**DOI:** 10.1007/s00701-025-06657-7

**Published:** 2025-09-02

**Authors:** Luca Zanuttini, Elisa Colombo, Grazia Menna, Victor E. Staartjes, Tristan van Doormaal, Niklaus Krayenbühl, Luca Regli, Carlo Serra

**Affiliations:** 1 Machine Intelligence in Clinical Neuroscience & Microsurgical Neuroanatomy (MICN) Laboratory, Department of Neurosurgery, Clinical Neuroscience Center, University Hospital Zurich, University of Zurich, Zurich, Switzerland; 2 Department of Biomedical and Neuromotor Sciences (DIBINEM), University of Bologna, Bologna, Italy; 3 Department of Neurosurgery, University Hospital Zürich, Zurich, Switzerland; 4 Department of Neurological Surgery, Fondazione Policlinico Universitario Agostino Gemelli, Rome, Italy; 5 Department of Neurosurgery, University Medical Center Utrecht, Utrecht, Netherlands; 6 Department of Neurosurgery, University Children‘s Hospital Zürich and University of Zürich, Zurich, Switzerland

**Keywords:** Thalamic glioma, MxR, Microneurosurgery, Thalamus

## Abstract

**Background:**

Microsurgical resection of thalamic tumors requires precise anatomical knowledge and meticulous preoperative planning. Given the complexity of thalamic surgery, selecting an optimal surgical approach demands an accurate three-dimensional understanding of relevant structures. Advanced imaging post-processing, including three-dimensional (3D) model construction, can aid surgical planning and mental rehearsal of the procedure. The integration of Mixed Reality (MxR) with interactive holograms may further enhance anatomical clarity, improve risk assessment, and facilitate safer and more extensive tumor resection.

**Methods:**

This retrospective study analyzed patients who underwent thalamic tumor resection with holographic surgical planning between 2022 and 2024. Key anatomical structures, particularly those delimiting the four “free thalamic surfaces”, in contact with cerebrospinal fluid spaces, were segmented using post-processing software (Lumi and 3D Slicer) based on volumetric MRI sequences. The resulting patient-specific holograms were utilized preoperatively to simulate and select the optimal surgical approach and intraoperatively to verify its suitability. Demographic, clinical, radiological, and perioperative data were collected.

**Results:**

Ten surgical procedures were performed in nine patients, including seven neuroepithelial tumors and two metastases. All critical structures were successfully segmented, allowing effective surgical simulation and approach selection (8 Anterior Interhemispheric Transcallosal, 2 Perimedian Supracerebellar Transtentorial approaches). The additional planning time for segmentation averaged 45 min. The mean extent of resection achieved was 94.88% (range: 78.6%–100%). At 3-months postoperative follow-up one out of nine patients experienced a permanent new neurological deficit due to surgery.

**Conclusion:**

Integrating MxR into the preoperative workflow for microsurgical removal of complex thalamic tumors proved to be a valuable tool for surgical planning, risk assessment, and intraoperative guidance. The additional preparation time required for holographic simulation appears justified given the anatomical complexity of thalamic lesions.

## Introduction

 From a functional point of view, the thalamus is an anatomical structure of key importance, as it concentrates highly relevant neurological functions in a relatively small volume. From a surgical perspective, the complexity of the region is further compounded by its deep location, which significantly restricts access, thus rendering it a high-risk region for surgical intervention [11, 26, 27]. Moreover, from a topographical standpoint, the thalamus lies adjacent to several highly eloquent telencephalic, diencephalic and mesencephalic structures. The thalamus is also characterized by a highly variable vascular supply most commonly coming from deep perforating branches of the posterior cerebral artery (PCA) and the posterior communicating artery (PCoA) [17, 30]. This variability makes the consequences of arterial injury largely unpredictable, ranging from minor to highly devastating [9, 25, 31]. Venous drainage also shows marked anatomical variability and is primarily provided by tributaries of the internal cerebral vein and the basal vein of Rosenthal [10].

 Nevertheless, micro-surgical removal of thalamic tumors is a feasible treatment option, with documented satisfactory outcomes. Adequate neuroanatomical knowledge and microsurgical technique are essential for determining the most appropriate surgical approach, and for its successful planning and execution [19, 20, 28].

Of the various steps involved in surgically treating a thalamic lesion, choosing and planning the surgical approach plays a much more important role than it does in other types of surgery, as outlined above. The surgeon is required to a) make an accurate topographical diagnosis of the lesion and b) accurately conceptualize it in three dimensions, along with the surrounding anatomy, so as to be able to c) choose the most suitable surgical approach. This step is arguably pivotal in the training of every neurosurgeon, particularly in relation to the learning curve of complex surgical procedures.

 Until recently, the choice and planning of surgical approaches primarily relied on conventional two-dimensional (2D) imaging. While this approach may provide sufficient information for an experienced neurosurgeon on a routine surgical case, it may not be sufficient for neurosurgeons confronted with a rare or unusual lesion, such as a thalamic or other deeply located lesions [32]. A precise and accurate three-dimensional representation of the patient's anatomy can facilitate informed decision-making and surgical planning, enhancing the surgeon's confidence during operations [1, 2, 12].

 The digitalization of the medical field has led to significant advancements in imaging modalities, including the emergence of augmented reality (AR) and, more specifically, mixed reality (MR). By combining real-world visual perception with virtual, interactive holographic projections, MxR enables surgeons to manipulate and interact with 3D reconstructions of patient-specific anatomy derived from preoperative imaging [1, 3]. Three-dimensional interactive holographic models allow neurosurgeons at all levels of experience to examine complex relationships between the tumor, and adjacent brain structures from multiple perspectives before the surgery. Unlike conventional 2D imaging, MxR supports a more intuitive understanding of spatial configurations, enhancing the surgeon’s ability to anticipate challenges, plan precise trajectories and minimize brain retraction [4, 23]. Therefore, MxR may represent a great perioperative adjuvant to the microsurgical technique, particularly in such complex cases as those related to thalamic surgery.

This study presents an analysis of a single-center, single-operator series of thalamic tumor resections supported by preoperative MxR visualization technology. The analysis aims to demonstrate the usefulness of MxR in this context and provide insight into its integration with established microsurgical techniques.

## Methods and materials

### Clinical data

 A retrospective analysis was conducted using prospectively collected data on patients who underwent surgical procedures for a thalamic lesion at our institution between 2022 and 2024 with the support of mixed reality (MxR). All patients were operated by a single surgeon (C.S.). All patients had preoperative and early postoperative (less than 48 h) 3 T brain magnetic resonance imaging. Volumetric measurements were performed on manual or semi-automatic tumor segmentations in the 3D slicer software [6]. Extent of resection (EOR) was evaluated by comparing preoperative and postoperative volumetry as commonly described [14]. An EOR of greater than 99% was defined as a Gross Total Resection (GTR), while a Near-Total Resection (NTR) was classified as an EOR between 99 and 95%, and a Subtotal Resection (STR) was defined as an EOR of less than 95%. All patients underwent thorough neurological examination at admission, postoperatively (< 6 h postoperative), at discharge, and at the three-month postoperative control. The follow-up period and postoperative treatment was contingent upon the histopathology of the lesion. New-onset postoperative neurological deficits were considered permanent when present at the three-month postoperative follow-up. Descriptive statistics were employed to report patients'data. Written informed consent was obtained from all patients. This study was made in accordance with the ethical standards set forth in the Declaration of Helsinki and approved by the institutional review committee.

### Surgical planning with mixed reality system

**Fig. 1:**
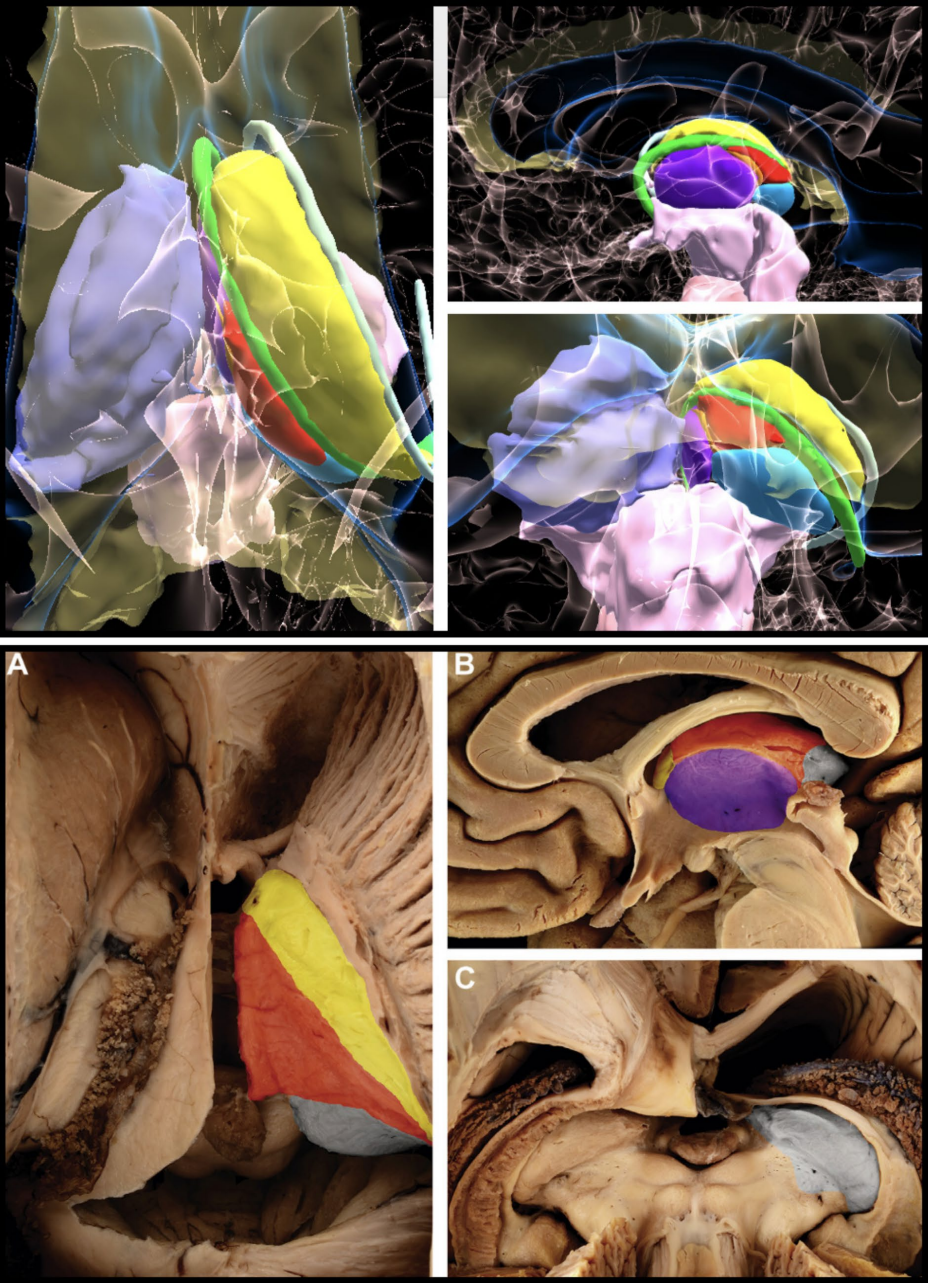
Holographic representation of the free thalamic surfaces and relevant anatomical landmarks. The appearance of the holograms (upper panel) is compared to that of the thalamic surfaces on a specimen (lower panel) to highlight the models'fidelity in neuroanatomical topography. Red indicates the velar surface; purple, the third ventricle surface; light blue, the cisternal surface; and yellow, the lateral ventricle surface. The lateral ventricle surface is located between the stria terminalis, laterally, and the taenia choroidea, medially. The margins of the third ventricle surface are defined by the stria medullaris thalami, on its superior and on most of its anterior and posterior borders. The remainder of the posterior border is defined, in a cranial-to-caudal direction, by the habenular commissure, the pineal recess, and the posterior commissure. The hypothalamic sulcus demarcates the inferior border. The cisternal surface corresponds to the part of the pulvinar medial to the fimbria and medial and lateral geniculate bodies. The lateral border is defined by the taenia choroidea. Superomedially, the cisternal surface continues into the velar surface above the habenular commissure. Below the habenular commissure, the medial border continues in the lateral margin of the pineal recess and the posterior commissure. The velar surface is located between the taenia choroidea and the stria medullaris thalami. The stria terminalis is represented in white; the fornix in green; and the stria medullaris in orange. The tenia choroidea is not represented as the fornix serves, also is the microsurgical view, as a landmark for its course. Dissection pictures reprinted from World Neurosurgery, Volume 97, pages 438–452 (Serra C, Türe U, Krayenbühl N, Şengül G, Yaşargil DCH, Yaşargil MG. Topographic classification of the thalamus surfaces related to microneurosurgery: a white matter fiber microdissection study, Copyright 2017), with permission from Elsevier under the STM guidelines

 All lesions were approached through the so-called ‘free thalamic surface’, defined as the portion of the thalamus that borders cerebrospinal fluid (CSF) spaces, making it accessible via purely transcisternal (TCi) or transcallosal-transventricular (TCTV) surgical corridors. This enables surgical access while minimizing manipulation of otherwise healthy cortical and white matter structures [27, 28]. For surgical purposes, the thalamic free surface can be further subdivided according to clearly recognizable anatomical landmarks into four surfaces: the velar surface, the third ventricle surface, the lateral ventricle surface and the cisternal surface [27] (Fig. 1). These anatomical landmarks are clearly recognizable even on standard neuroimaging and therefore allow the identification of the free thalamic surface closest to the tumor. Each of these surfaces can be reached with one or more TCi/TCTV approaches. The final choice also depends on other concomitant factors, the most important of them being the relation of the tumor with the splenium of the corpus callosum and with the deep venous system of the transverse fissure, as well as the presence of CSF circulation disturbances as previously detailed [28]. The need for 3 T-ioMR may also play a role. It is difficult to provide an algorithmic guide for the approach to individual surfaces since each case can present unique variations. In general, it can be said that the *lateral ventricle surface* should be approached preferentially with an anterior interhemispheric transcallosal (AIT) approach, particularly in cases of concomitant hydrocephalus. Similarly, the *velar surface* can be approached preferentially via an AIT approach, especially if the choroidal fissure has already been opened up by the growing tumor. The *third ventricle surface* can be approached either through the foramen of Monro after an AIT approach, as in the case of concomitant hydrocephalus, or posteriorly, with a perimedian contralateral supracerebellar suprapineal approach (PeCSS). The cisternal surface can be approached in two ways: either via a posterior interhemispheric transtentorial approach (PITS) if splenic and venous anatomy allows for it, or via a perimedian supracerebellar transtentorial approach (PeST).

**Fig. 2:**
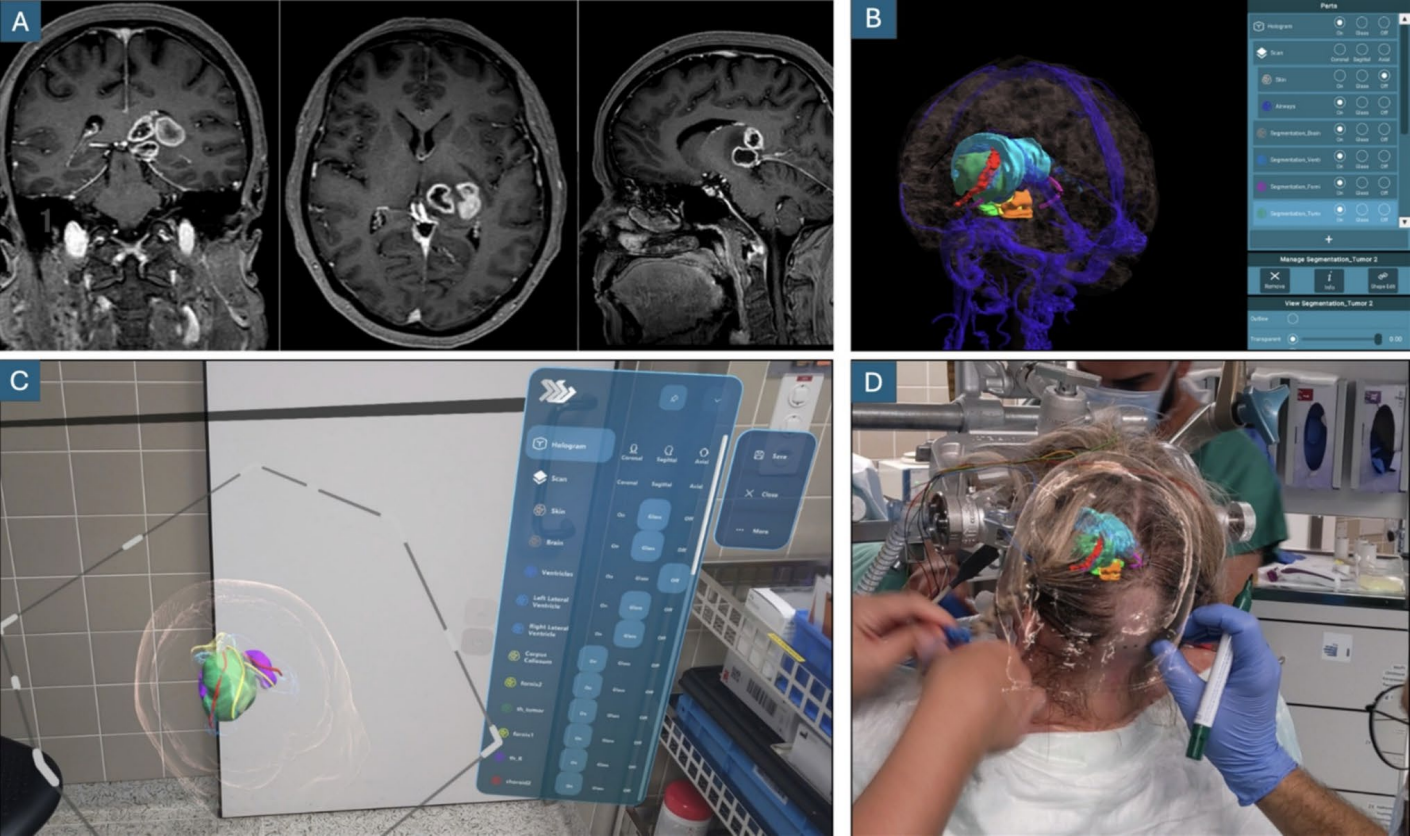
Steps of the MxR-aided preoperative planning. 2-D standard imaging along the common planes of a left thalamic glioma confined to the pulvinar (Panel A). DICOM images are loaded onto the MxR cloud where a 3-D model is created (panel B). Relevant structures are segmented either semi-automatically or manually, depending on the complexity of the scenario. The three-dimensional model obtained can then be explored in a virtual reality environment to better understand the topographical anatomy of the tumor in relation to the free thalamic surfaces and the most relevant anatomical structures previously listed in the text (panel C). In this phase, the surgical approach is simulated and then chosen. Finally, the model can be viewed on a head-up display even during surgery and used to optimize patient positioning and surgical incisions (panel D)

 For each patient, three-dimensional models of the tumor and relevant neuroanatomical structures were generated from preoperative MRI data. The following structures were segmented for each case: tumor, fornix, choroid plexus, stria terminalis, stria medullaris thalami, ventricles, corpus callosum, deep venous system, brain, and cerebellum. Segmentation was performed using a combination of automated and, when necessary, manual or semi-automated techniques. Automated segmentation was performed using a validated mesh-based algorithm (Disior) integrated within the cloud-based Lumi platform (Augmedit BV, Naarden, The Netherlands) [1, 8], which enabled rapid model generation from DICOM files. In cases where the quality of the generated model was deemed insufficient, or when segmenting small and anatomically complex structures not covered by the algorithm, manual or semi-automatic segmentation was carried out using 3D Slicer (version 5.6) [6]. Larger structures such as ventricles or tumors, when not accurately segmented automatically, were segmented semi-automatically using the “Grow from Seeds” module. Thin tubular structures, such as the stria terminalis and stria medullaris thalami, were segmented manually using the “Draw Tube” module from the SegmentEditorExtraEffects extension. Final 3D models were uploaded to the Lumi platform and visualized perioperatively using mixed reality glasses (HoloLens 2, Microsoft, Redmond, Washington, USA) (Fig. 2).

The day before surgery, the patient-specific anatomy and topography of the pathology are studied using this 3D interactive modality. The anatomical relationships between the tumor and the four free thalamic surfaces are studied in detail, as are the relationships between the tumor and the other anatomical structures previously mentioned. These relationships are examined in both general terms and in terms specific to each individual approach. Each approach can then be simulated in an immersive way, allowing the pros and cons to be evaluated. Then, the best approach is chosen based on the MxR simulation.

Finally, in the operating room, a manual registration between the hologram and the patient's head is performed during the non-sterile intraoperative phase after head fixation using reliable surface landmarks (nasion, inion, and tragus). The correctness of the hologram-head matching is then verified by means of standard navigation. Any modification of the patients/head positioning is noted. Intraoperative hologram visualization allows for adjustment of the patient's position to achieve optimal surgical angles. This is particularly important for deep surgical corridors, such as those required for thalamic surgery. Once the patient has been positioned, the skin incision can be adapted with the assistance of direct 3D visualization of the deep target and surrounding structures.

## Results

 A total of 10 surgical procedures for the removal of a thalamic lesion were performed on 9 patients (M:F = 4:5) with a mean age of 48 years (range 22–73) during the study period. The goal was to achieve the maximum safe resection. Details of clinical presentation are reported in Table 1. Two of the lesions were metastases, one was a low-grade glioma, and the remaining six were high-grade gliomas, two of which exhibited a H3 mutation. One patient had previously undergone a stereotactic biopsy, while one other patient underwent two surgical procedures for cytoreduction during the same hospitalization. The first procedure was performed in an emergency setting to lower intracranial pressure, and the second was for oncological purposes. An AIT approach was selected for 8 surgical procedures; in the remaining 2 cases a PeST approach was chosen.

**Table 1:** Clinical and demographic characteristics of the study population. LGG = low grade glioma; HGG = high grade glioma; KPS = Karnofsky Performance Status; FU = follow-up; ICU LOS = intensive care unit length of stay; AIT = anterior interhemispheric transcallosal approach; PeST = perimedian supracerebellar transtentorial approach; GNT = glioneuronal tumor; DHGG NEC = diffuse high-grade glioma not elsewhere classified; H3.K27M/H3.G34M = histone H3 mutations K27M/G34M; H3wt = histone H3 wild-type; EOR = extent of resection; GTR = gross total resection; STR = subtotal resection; N/A = not applicable

Sex, no (%)	
M	4
F	5
Mean age in yrs (median, range)	48 (52, 22–73)
Age group, no. (%), LGG, HGG	
Pediatric: age < 18yrs	0
Adults: age > 18yrs	9
Side of surgery, no. (%)	
Rt	8
Lt	2
No. of prior operations for oncological purposes (no. of patients)	
0	5
1	4
Clinical outcome	
Mean KPS (range)	
At admission	74 (40–90)
At discharge	71 (40–100)
At 3-mo FU	75 (50–100)
KPS deterioration, no. (%)	
At discharge	3
At 3-mo FU	1
KPS improvement, no. (%)	
At discharge	2
At 3-mo FU	3
Medical complication, no. (%)	
CSF infection and epidural abscess	1
Neurological outcome	
New/worsening of hemiparesis, no. (%)	
At discharge	5
At 3-mo FU	0
Improvement/resolution of hemiparesis, no./total no. (%)	
At discharge	0
At 3-mo FU	5
Improvement of preoperative aphasia at 3-mo FU, no	1
Mean ICU LOS, days (median, range)	2 (2, 1–5)
Mean hospitalization length, days (median, range)	13.2 (11, 4–38)
Approaches:	
AIT	8
PeST	2
Histopathology:	
Rosette-forming GNT	1
IDHwt Glioblastoma	2
DHGG NEC	1
Diffuse midline glioma H3.K27M	1
Hemispheric glioma H3.G34M	1
Diffuse pediatric-type HGG H3wt IDHwt	1
Metastasis	2
WHO grade:	
I	1
IV	6
N/A	2
Volumetry:	
Mean EOR	94.88%
Mean preop lesion vol (cm3)	34.17
Mean residual lesion vol (cm3)	3.77
EOR:	
GTR	6
NTR	0
STR	3

### Clinical and surgical outcome

The mean preoperative tumoral volume was 34.17 cc, while the mean residual tumor volume was 3.77 cc, with a mean extent of resection (EOR) of 94.88%. In 6 out of 9 patients, an Extent of Resection (EOR) of > 95% was achieved, with 6 patients exhibiting a Gross Total Resection (GTR). In the remaining 3 cases a STR resection was achieved.

The median length of stay in the ICU was 2 days, while the median length of hospitalization was 11 days. At the time of discharge, 5 patients exhibited new-onset or worsening of hemiparesis. However, all patients who had a 3-month postoperative neurological examination demonstrated an improvement, with two patients exhibiting complete resolution. Overall, one patient exhibited a new-onset, permanent, mild hemiparesis as a consequence of surgery. In a single case, there was a three-month postoperative worsening of hemiparesis in a patient who did not present with any new-onset postoperative neurological deficits. This was indicative of an early tumor recurrence of a WHO grade IV glioma. As with the neurological deficits, the mean KPS decreased at discharge (74 at admission to 71), but showed a recovery at the 3-month follow-up (75). There was one mortality only, related to one young patient presenting in coma with bilateral mydriasis who underwent emergent surgery. Unluckily he never recovered from coma and magnetic resonance imaging (MRI) showed bilateral midbrain infarction possibly due to acute herniation by tumor compression. Death occurred in the fourth postoperative day. One patient died before the 3-month follow-up control because of an early recurrence. One patient experienced a medical complication: a CSF infection with subsequent epidural abscess formation and drug-induced hepatopathy secondary to antibiotic treatment. This underwent further surgical procedures for abscess evacuation and operculum removal and, six months later, for a custom-made cranioplasty. There were no infection-related new-onset neurological deficits. Four patients underwent a total of 6 procedures for CSF diversion. Three patients underwent external ventriculostomy and one endoscopic third ventriculostomy (3VC), all prior to thalamic surgery to relieve intracranial hypertension due to tumor-related obstructive hydrocephalus. In one case a 3VC was performed at the end of tumor removal through an AIT approach. In all cases, hydrocephalus was resolved by the time of hospital discharge. However, in one patient, a delayed VP shunt positioning was required despite the prior 3VC.

### Implementation of mixed reality and cases presentation

In the preoperative phase, MxR was used to evaluate three-dimensional relationship between the tumor, the free thalamic surfaces and adjacent neural structures and to rehearse the chosen surgical approach. In the non-sterile phase of the surgery, following patient positioning and head fixation, the hologram was matched to the head of the patient. This was done to confirm patient and head positioning and to tailor the skin incision, as it would have been done with the support of a neuronavigation system in a standard neurosurgical environment. The mean required processing time was 45 min, including hologram generation and patient registration.

The following four cases of thalamic gliomas are presented: one for each free thalamic surface involvement. For each case, standard 2D imaging is presented along with 3D models of relevant neuroanatomy, and the relevant factors for the decision-making process in the preoperative planning are exposed.

**Fig. 3:**
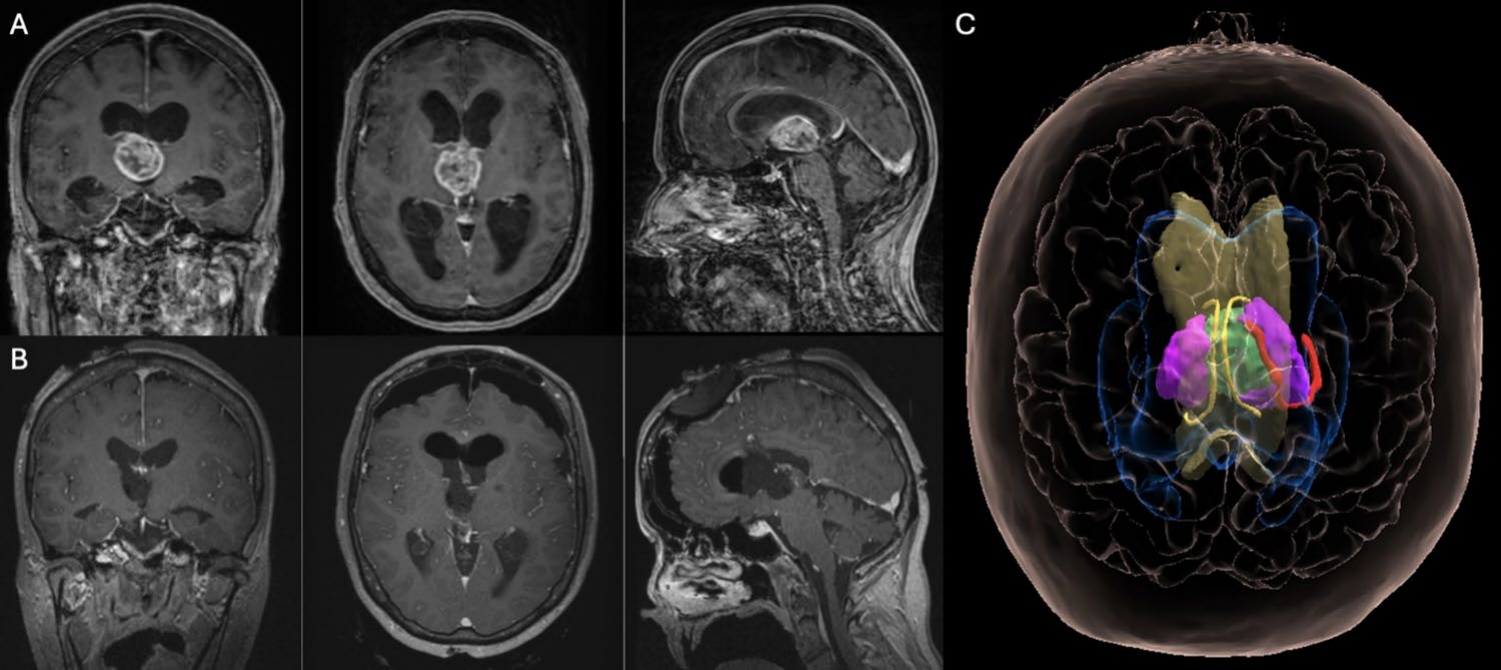
Pre- (panel A) and postoperative (panel B) coronal, axial and sagittal contrast-enhanced T1-weighted MRI of a patient harboring a glioma emerging from the velar surface of the thalamus. The three-dimensional reconstruction in MxR (panel C) clearly show the widening of the choroidal fissure due to the tumor, which prompts a removal via the cella media of the lateral ventricle, easily reachable via a AIT approach. Color legend: bright yellow, fornix; red, choroidal plexus; violet, right and left thalamus; green, tumor; translucent yellow, corpus callosum; blue, lateral ventricles
  A 67-year-old female patient presented with neurocognitive deficits, including anterograde amnesia and spatial and temporal disorientation. The MRI appearance and 3D models demonstrate a thalamic tumor abutting the velar surface of the right thalamus. The relationship between the tumor and the velar surface can be determined by observing the widening of the ipsilateral choroidal fissure, which creates a gap between the ipsilateral taenia fornicis and taenia thalami. This dynamic is exemplified by the lateral displacement of the plexus. This indicates that the tumor has grown upward from the velar surface, reaching the lateral ventricle. The tumor's location in the cella media of the lateral ventricle, when combined with the presence of radiological evidence of hydrocephalus, facilitates its accessibility via AIT. The histological and molecular report indicated a diffuse high-grade glioma, not elsewhere classified (NEC). The patient exhibited slight left hemiparesis at the postoperative neurological examination, which fully recovered at the 3-month follow-up. Neurocognitive deficits demonstrated signs of improvement, though the full restoration of previous levels was not observed.
- Case 2: approach to the cisternal surface via PeST (Fig. 4)

- Case 1: approach to the velar surface via AIT (Fig. 3)

**Fig. 4:**
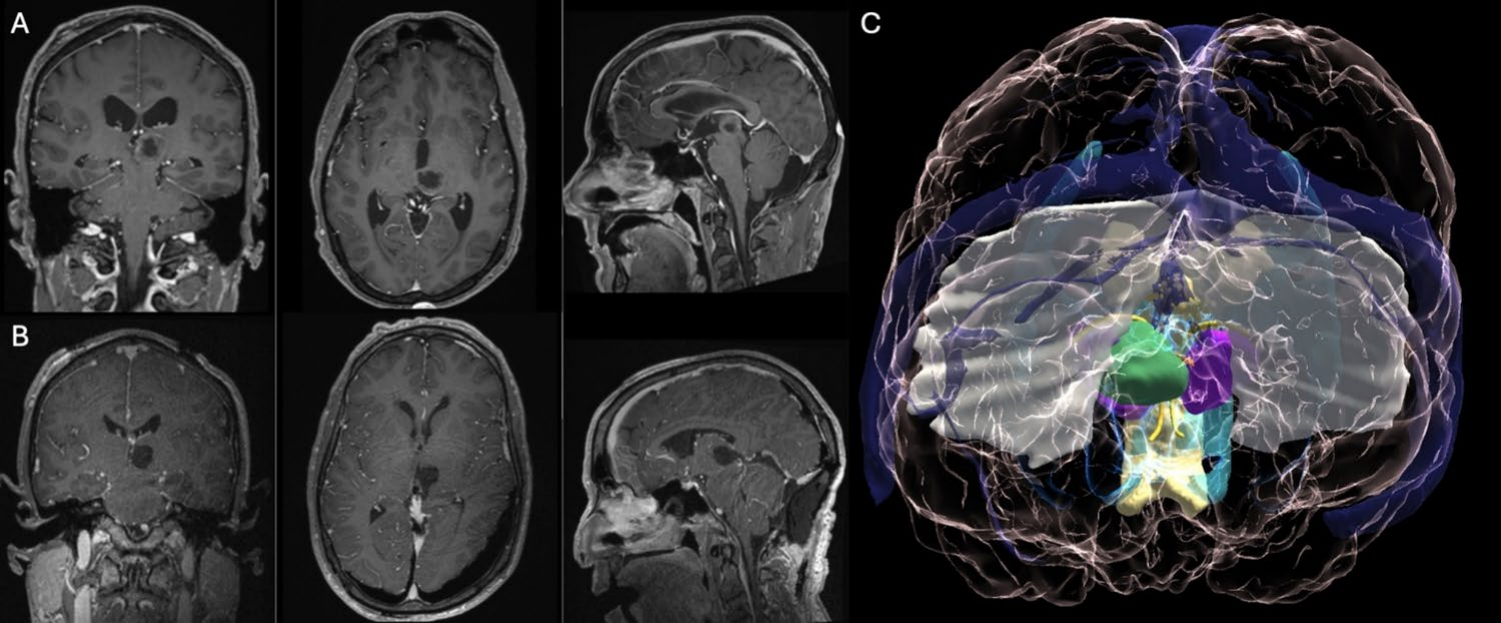
Pre- (panel A) and postoperative (panel B) coronal, axial and sagittal contrast-enhanced T1-weighted MRI of a patient harboring a glioma emerging from the cisternal surface of the thalamus. The imaging reveals that the lesion abuts the quadrigeminal cistern. Additionally, it demonstrates that the splenium appears to cover the dorsal surface of the lesion. The simulation of the PeST approach in MxR (panel C) clearly shows how this approach permits an optimal view and reachability of the lesion avoiding any manipulation of the splenium. Color legend: bright yellow, fornix; violet, right and left thalamus; green, tumor; translucent yellow, corpus callosum; light blue, lateral ventricles; dark blue, dural sinuses; grey, tentorium
  A 52-year-old male presented with neurocognitive deficits. Preoperative MRI and 3D models revealed a tumor in the left thalamus abutting its cisternal surface. As depicted in the hologram, the tumor is visible as it emerges from the cisternal surface of the thalamus, medial to the ipsilateral fornix. The lesion extends upward along the inferior surface of the splenium of the corpus callosum. This feature renders a PITS approach unfeasible and prompts a PeST approach, which was performed in a lateral oblique position to allow for intraoperative MRI. A gross total removal was achieved. After histopathological analysis, the lesion was confirmed to be a lung adenocarcinoma metastasis. The patient's surgery was uneventful, and no new neurological deficits were experienced at discharge or during the 3-month postoperative follow-up.
- Case 3: approach to the third ventricles via AIT (Fig. 5)

**Fig. 5:**
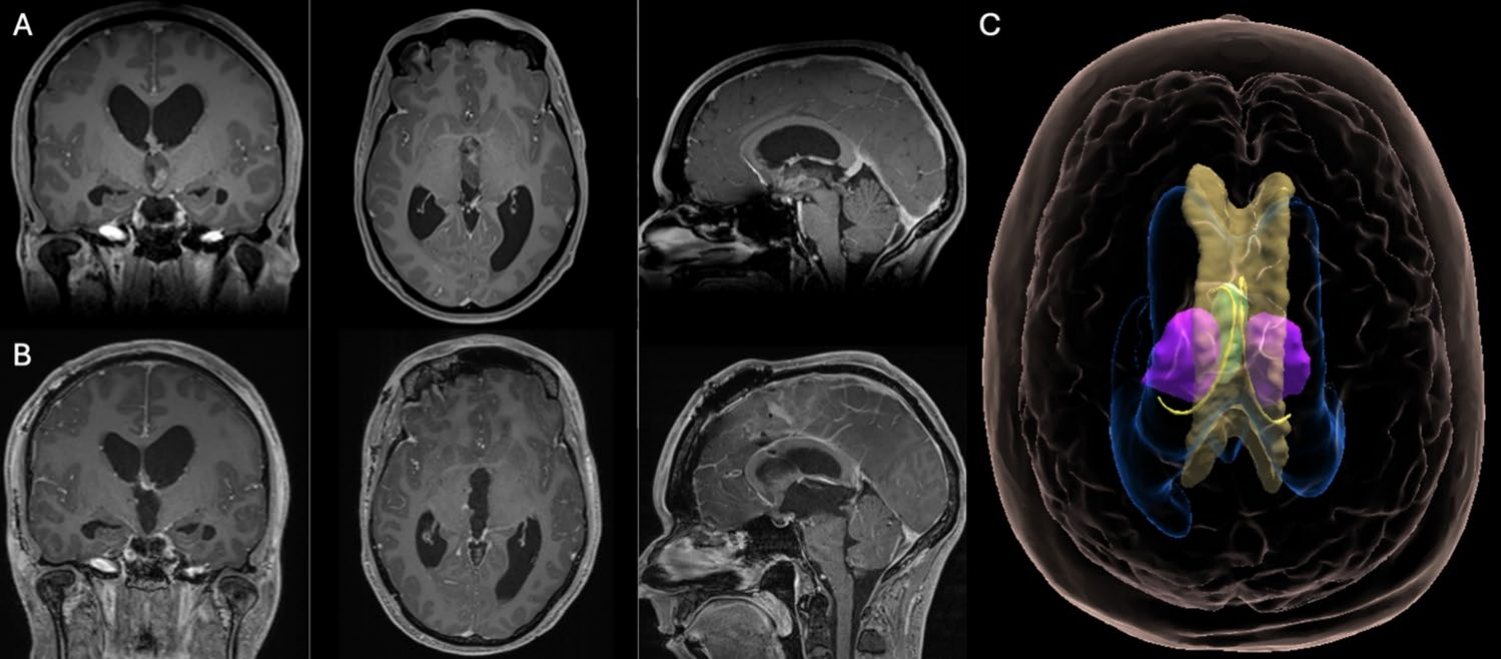
Pre- (panel A) and postoperative (panel B) coronal, axial and sagittal contrast-enhanced T1-weighted MRI of a patient harboring a glioma emerging from the third ventricle surface of the thalamus. The imaging shows that the lesion fills the third ventricle, thereby causing hydrocephalus and dilatation of the Foramen of Monro, as clearly indicated in MxR (panel C). The left fornix is significantly displaced caudomedially by the tumor. Color legend: bright yellow, fornix; violet, right and left thalamus; green, tumor; translucent yellow, corpus callosum; blue, lateral ventricles
  A 43-year-old male presented with gait apraxia. The MRI revealed a thalamic tumor that abutted the third ventricle surface of the left thalamus. In this case, the tumor mass, in conjunction with the hydrocephalic condition, contributed to the widening of the foramina of Monro. These factors enabled the performance of a resection through an AIT approach from the right side. Gross total resection was achieved. A histological examination revealed a rosette-forming glioneuronal tumor. The patient exhibited slight left hemiparesis at the postoperative neurological examination, which fully recovered at the 3-month follow-up.
- Case 4: approach to the lateral ventricles surface via AIT (Fig. 6)

**Fig. 6:**
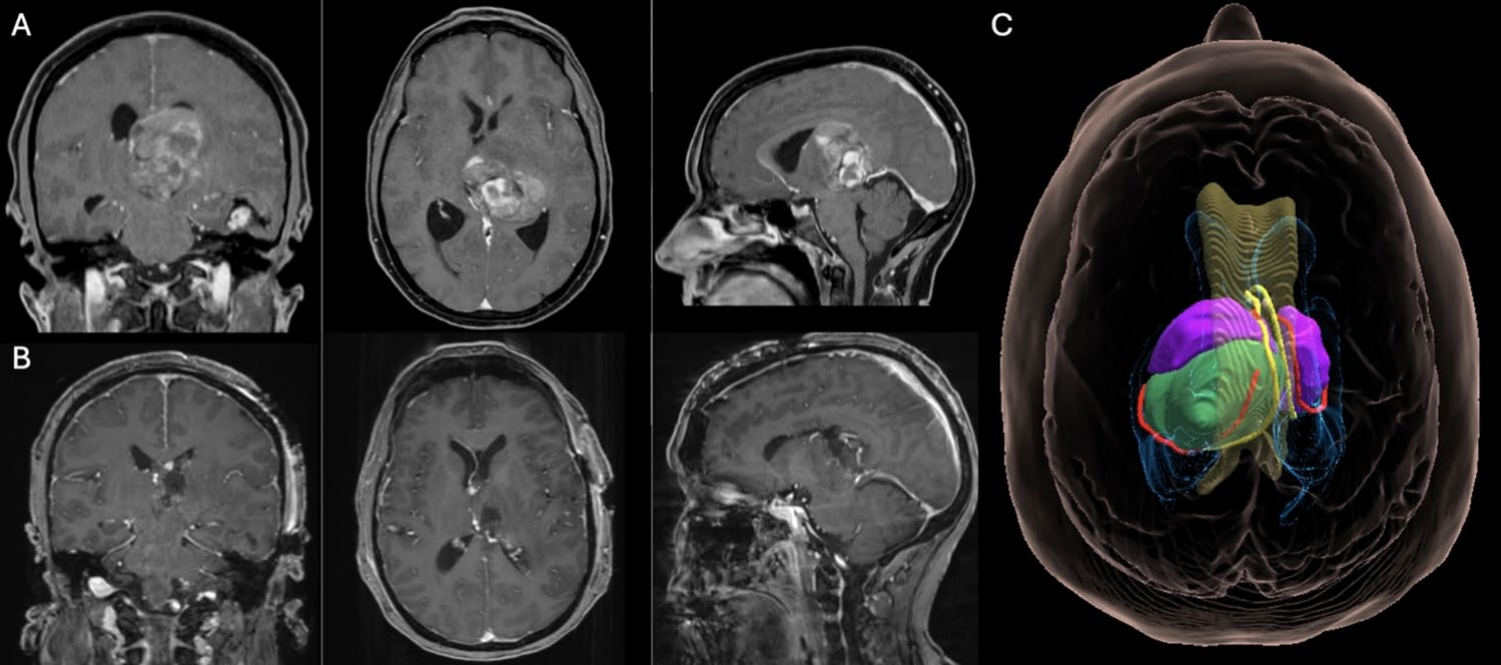
Pre- (panel A) and postoperative (panel B) coronal, axial and sagittal contrast-enhanced T1-weighted MRI of a patient harboring a large thalamic metastasis emerging via the lateral ventricle thalamic surface into the left lateral ventricle. This important topographic information can be inferred by observing the medial displacement of the fornix and the taenia thalami, which can be determined by the position of the choroid plexus. The MxR model (panel C) allows also preoperative simulation and validation of the angle of the approach. Color legend: bright yellow, fornix; red, choroidal plexus; violet, right and left thalamus; green, tumor; translucent yellow, corpus callosum; blue, lateral ventricles
  A 53-year-old female presented with motor aphasia and slight right hemiparesis. Imaging revealed a thalamic tumor abutting the lateral ventricle surface of the left thalamus, as well as a tumor located in the left inferior temporal gyrus (not depicted in figure). During the same surgical procedure, a left temporal craniotomy was performed to remove the temporal lesion, and then a right AIT approach was used to remove the left thalamic lesion. During the procedure, the patient's head was carefully repositioned from a lateral to a neutral stance between the two surgical phases. The selection of the AIT approach based solely on two-dimensional imaging is not straightforward due to the absence of hydrocephalus. However, MxR simulation provides reassurance to the surgeon when choosing AIT by demonstrating how the tumor emerges at the lateral ventricular surface of the thalamus. Additionally, it allows for the simulation and confirmation of the angle of approach to the tumor. The total removal of both lesions was successfully achieved. A pathological analysis revealed metastatic melanoma in both lesions. A postoperative neurological examination revealed a slight worsening of the pre-existing right hemiparesis. At the three-month postoperative follow-up, the patient demonstrated significant progress in both hemiparesis and motor aphasia, indicating notable improvement compared to their preoperative state.

## Discussion

 To the best of the authors'knowledge, this analysis is the first study to support the incorporation of MxR into the surgical planning and perioperative preparation for micro-surgical removal of thalamic tumors. The documented, satisfactory surgical outcomes at discharge and at the three-month follow-up period confirm the findings of the literature, which show that, provided adequate microneurosurgical principles, thalamic surgery is feasible and safely reproducible [5, 20, 22, 28, 33].

The study's key finding is the illustration of how MxR tools can be used in thalamic surgery. This finding underscores their value in diagnosing the topography of thalamic tumors in relation to thalamic surfaces. It also demonstrates their effectiveness in conceptualization, which effectively becomes a three-dimensional visualization of these tumors in relation to the surrounding anatomical structures. Furthermore, MxR tools can be used to simulate and validate the most suitable surgical approach. We believe that these advantages can help shorten surgical learning curves and, together with the essential principles of microneurosurgery, make these surgical approaches accessible to a wider audience of neurosurgeons and, therefore, patients.

### Thalamic surgery

 The surgical outcomes in this series, showing a mean extent of resection (EOR) of 94.88% and GTR achieved in two-thirds of cases, align with recent literature about thalamic tumors [5, 7, 18, 28]. The low rate of permanent complications (1 out of 9 cases) at 3-months postoperative follow-up and the recovery of preoperative Karnofsky Performance Status (KPS) in most patients reflect the safety, and thus feasibility, of such treatment option. In accordance with microneurosurgical principles, we exclusively employ transcisternal approaches, which provide direct access to the free surfaces of the thalamus while minimizing disruption to healthy cortical and subcortical structures. Although robust empirical data demonstrating superior outcomes compared to transcortical approaches remains to be published, rational analysis suggests that avoiding unnecessary damage to healthy brain tissue is a sensible choice to reduce the risk of postoperative morbidity. Our results appear to support this rationale. Obstructive hydrocephalus is a critical factor in the management of thalamic tumors, given its frequent presentation in this population [20, 22, 28]. In this series, 44% of patients required transient preoperative cerebrospinal fluid (CSF) diversion due to obstructive hydrocephalus, while 11% only required delayed ventriculoperitoneal shunting after surgery. These rates are comparable to the 5–6% postoperative hydrocephalus observed in larger cohorts [22].

### MxR in surgery

 Microsurgical resection of tumors demands meticulous planning and execution, particularly in highly critical areas surrounded by critical neurovascular structures and functionally significant pathways [26, 27]. Traditional methods of surgical planning, primarily relying on 2D imaging sequences, provide essential information but lack the ability to intuitively convey intricate 3D spatial relationships. Interestingly, the importance of a more realistic visualization of the surgical anatomy was already acknowledged by Professor Yaşargil, who used to flip side to side radiological images and even turn them upside down in order to visualize the correct lateralization and mimic the intraoperative angle of view. MxR, using patient-specific holograms, represents a transformative advancement in neurosurgical planning by addressing these limitations [1, 12]. One of the most significant advantages of MxR is the interactive 3D visualization of patient-specific anatomy. MxR, providing a tangible holographic representation of the tumor and surrounding structures, reduces the cognitive burden associated with mentally transforming data from standard 2D imaging into 3D spatial awareness. This allows the surgeon to focus more effectively on the surgical plan [13]. In addition, the implementation of 3D visualization tools has the potential to reduce the learning curve and increase the number of surgeons who are able to perform even the most complex cases [21, 23].

 The ability to manipulate the holographic model (rotating, zooming, and visualizing each structure selectively) offers a dynamic perspective that is unattainable with standard imaging techniques or navigation systems. This in turns enhances risk assessment and surgical preparation by enabling a close-to-reality preoperative rehearsal of the procedure, simulating surgical approaches and identifying optimal trajectories [1] *.* From a practical standpoint, MxR facilitates a more informed decision regarding the surgical approach during the preoperative phase. During the nonsterile intraoperative phase, it enables the adaptation of the patient's positioning and the skin incision design to the planned operative trajectory.

### MxR in thalamic surgery

 The use of virtual reality or mixed reality methods in neurosurgery is not in itself a disruptive innovation and has been a known option for almost thirty years [16, 29]. To date, the main limitation to the implementation of MxR methods in neurosurgery has always been the prolonged time required for preoperative image processing and segmentation, which is often incompatible with clinical routine.

 Indeed, at approximately 45 min, the average processing time in our series could be considered excessive and unjustified, even considering the expected benefits of MxR methods in terms of anatomical understanding and surgical planning. However, in our opinion, the situation changes when MxR is applied to more complex surgeries, such as thalamic surgery. In this case, the anatomical and planning complexities can be discouraging. In fact, most neurosurgeons are still discouraged by it to the point that they often consider such lesions inoperable, despite several authors postulating the feasibility of such surgery in the 1980 s [15, 34].

 As with other highly complex surgeries, the scientific community faces the issue of reproducibility in thalamic surgery, which certainly decreases as complexity increases. One response may be to declare a condition inoperable or more suitably treatable by other methods. However, one could also argue for simplifying the most effective treatments or training more surgeons to perform them, regardless of their complexity. Regarding gliomas, there is definitive evidence that EOR significantly correlates with oncological outcomes [24]. Therefore, any technological or scientific advance that allows the concept of maximal safe resection to be extended to the widest possible number of patients should be welcomed. This includes patients with lesions in highly critical regions. Considering this, we deem the additional 45 min required for preoperative image processing in our study a reasonable investment.

### Limitations

All procedures were performed by a single, senior surgeon at a single center, which may limit generalizability. However, the very aim of this study is to underscore the potential of MxR to improve the spatial understanding of deep-seated neuroanatomical structures and to support structured preoperative planning and mental rehearsal. By facilitating these processes, MxR could improve accessibility to such complex surgeries and promote their integration into broader neurosurgical practice.

The lack of a control group limits the ability to draw definitive conclusions about the added clinical value of MxR. Future studies including appropriate comparisons will be needed to better clarify its role.

Beyond these study-specific aspects, the implementation of MxR in the neurosurgical workflow is not without limitations. First, segmentation accuracy may vary depending on imaging quality and anatomical complexity, particularly for small or low-contrast structures. While this may affect visual fidelity, it is important to emphasize that the primary purpose of MxR planning is to provide a macroscopic and qualitative spatial understanding of tumor topography relative to key anatomical landmarks. In this context, manual refinements can often be limited or omitted when not essential for surgical planning, thereby reducing workload and improving efficiency. Second, MxR integration entails a learning curve, both in segmentation workflows and intraoperative interpretation; however, in our experience, this is not particularly steep, and once familiarized with the process, the advantages clearly outweigh the initial effort. Finally, although commercial platforms may involve licensing fees and specific hardware (e.g., HoloLens headsets), the growing availability of open-source imaging tools such as 3D Slicer and the decreasing cost of MxR head-mounted displays (HMDs) suggest that this technology may soon complement conventional navigation systems.

## Conclusion

This study represents the first application of MxR technology in the surgical workflow of thalamic microneurosurgery. Moreover, it highlights the potential of 3D MxR interactive visualization to provide neurosurgeons with a more precise preoperative surgical planning, rehearsal, patient positioning, and skin incision planning, thus supporting microsurgical technique, especially in highly demanding surgeries such as those involving thalamic tumors.

The present analysis supports the safety of microsurgical removal of thalamic lesions, with an acceptable level of morbidity. In our view, maximal safe microsurgical resection should be pursued even in such challenging locations, and every technical adjunct that may facilitate this goal deserves to be explored. Technologies such as MxR may contribute to making these procedures more accessible and reproducible. Future studies should quantitatively assess how MxR can enhance the safety and extent of tumor resection and promote its integration into routine surgical workflows.

## Data Availability

No datasets were generated or analysed during the current study.

## References

[1] Colombo E, Regli L, Esposito G et al (2023) Mixed reality for cranial neurosurgical planning: a single-center applicability study with the first 107 subsequent holograms. Oper Neurosurg. 10.1227/ons.0000000000001033 38156882 10.1227/ons.0000000000001033 PMC11008664

[2] Colombo E, Bektas D, Regli L, Van Doormaal T (2023) Case report: impact of mixed reality on anatomical understanding and surgical planning in a complex fourth ventricular tumor extending to the lamina quadrigemina. Front Surg 10:1227473. 10.3389/fsurg.2023.1227473 37675252 10.3389/fsurg.2023.1227473 PMC10477590

[3] Colombo E, Bektas D, Regli L, Van Doormaal T (2023) Case report: Impact of mixed reality on anatomical understanding and surgical planning in a complex fourth ventricular tumor extending to the lamina quadrigemina. Front Surg 10:1227473. 10.3389/fsurg.2023.1227473 37675252 10.3389/fsurg.2023.1227473 PMC10477590

[4] Colombo E, Lutters B, Kos T, Van Doormaal T (2023) Application of virtual and mixed reality for 3D visualization in intracranial aneurysm surgery planning: a systematic review. Front Surg 10:1227510. 10.3389/fsurg.2023.1227510 37829601 10.3389/fsurg.2023.1227510 PMC10564996

[5] Esquenazi Y, Moussazadeh N, Link TW et al (2018) Thalamic glioblastoma: clinical presentation, management strategies, and outcomes. Neurosurgery 83(1):76–85. 10.1093/neuros/nyx349 28973417 10.1093/neuros/nyx349 PMC6939410

[6] Fedorov A, Beichel R, Kalpathy-Cramer J et al (2012) 3D slicer as an image computing platform for the Quantitative Imaging Network. Magn Reson Imaging 30(9):1323–1341. 10.1016/j.mri.2012.05.001 22770690 10.1016/j.mri.2012.05.001 PMC3466397

[7] Ferroli P, Restelli F, Bertolini G et al (2023) Are thalamic intrinsic lesions operable? No-man’s land revisited by the analysis of a large retrospective, mono-institutional, cohort. Cancers (Basel) 15(2):361. 10.3390/cancers15020361 36672311 10.3390/cancers15020361 PMC9856718

[8] Fick T, Van Doormaal JAM, Tosic L et al (2021) Fully automatic brain tumor segmentation for 3D evaluation in augmented reality. Neurosurg Focus 51(2):E14. 10.3171/2021.5.FOCUS21200 34333477 10.3171/2021.5.FOCUS21200

[9] Freitas GRD, Bogousslavsky J (2002) Thalamic infarcts. In: Donnan G, Norrving B, Bamford J, Bogousslavsky J (eds) Subcortical Stroke. Oxford University Press, New York, NY, pp 255–286. 10.1093/oso/9780192631572.003.0021

[10] Giudicelli G, Salamon G (1970) The veins of the thalamus. Neuroradiology 1(2):92–98. 10.1007/BF00389441

[11] Hwang K, Bertolero MA, Liu WB, D’Esposito M (2017) The human thalamus is an integrative hub for functional brain networks. J Neurosci 37(23):5594–5607. 10.1523/JNEUROSCI.0067-17.2017 28450543 10.1523/JNEUROSCI.0067-17.2017 PMC5469300

[12] Isikay I, Cekic E, Baylarov B, Tunc O, Hanalioglu S (2024) Narrative review of patient-specific 3D visualization and reality technologies in skull base neurosurgery: enhancements in surgical training, planning, and navigation. Front Surg 11:1427844. 10.3389/fsurg.2024.1427844 39081485 10.3389/fsurg.2024.1427844 PMC11287220

[13] Jeffri NFS, Awang Rambli DR (2021) A review of augmented reality systems and their effects on mental workload and task performance. Heliyon 7(3):e06277. 10.1016/j.heliyon.2021.e06277 33748449 10.1016/j.heliyon.2021.e06277 PMC7969906

[14] Karschnia P, Young JS, Dono A et al (2023) Prognostic validation of a new classification system for extent of resection in glioblastoma: a report of the RANO*resect*group. Neuro-Oncol 25(5):940–954. 10.1093/neuonc/noac193 35961053 10.1093/neuonc/noac193 PMC10158281

[15] Kelly PJ (1989) Stereotactic biopsy and resection of thalamic astrocytomas. Neurosurgery 25(2):185–194; discussion 194–195. 2549442

[16] Kockro RA, Tsai YT, Ng I et al (2009) DEX-RAY: augmented reality neurosurgical navigation with a handheld video probe. Neurosurgery 65(4):795–808. 10.1227/01.NEU.0000349918.36700.1C 19834386 10.1227/01.NEU.0000349918.36700.1C

[17] Lazorthes G, Salamon G (1971) The arteries of the thalamus: an anatomical and radiological study. J Neurosurg 34(1):23–26. 10.3171/jns.1971.34.1.0023 5539644 10.3171/jns.1971.34.1.0023

[18] Li Z, Wu H, Wu B et al (2020) Long term follow-up and outcomes in adult patients with thalamic gliomas. Clin Neurol Neurosurg 195:105888. 10.1016/j.clineuro.2020.105888 32450499 10.1016/j.clineuro.2020.105888

[19] Martínez Santos JL, Aljuboori Z, Richardson AM et al (2024) Microsurgical anatomy and approaches to thalamic gliomas. Part 1: A cartography guide for navigating to the thalamus. Integrating 3D model rendering with anatomical dissections. J Neurosurg 141(6):1457–1471. 10.3171/2024.3.JNS232049 39029125 10.3171/2024.3.JNS232049

[20] Martínez Santos JL, Aljuboori Z, Richardson AM et al (2024) Microsurgical anatomy and approaches to thalamic gliomas. Part 2: Maximal safe resection of thalamic gliomas improves outcomes. A single-center experience. J Neurosurg 141(6):1472–1483. 10.3171/2024.3.JNS232067 39029116 10.3171/2024.3.JNS232067

[21] Mikhail M, Mithani K, Ibrahim GM (2019) Presurgical and intraoperative augmented reality in neuro-oncologic surgery: clinical experiences and limitations. World Neurosurg 128:268–276. 10.1016/j.wneu.2019.04.256 31103764 10.1016/j.wneu.2019.04.256

[22] Palmisciano P, El Ahmadieh TY, Haider AS et al (2021) Thalamic gliomas in adults: a systematic review of clinical characteristics, treatment strategies, and survival outcomes. J Neurooncol 155(3):215–224. 10.1007/s11060-021-03898-1 34797525 10.1007/s11060-021-03898-1

[23] Ragnhildstveit A, Li C, Zimmerman MH et al (2023) Intra-operative applications of augmented reality in glioma surgery: a systematic review. Front Surg 10:1245851. 10.3389/fsurg.2023.1245851 37671031 10.3389/fsurg.2023.1245851 PMC10476869

[24] Sanai N, Polley MY, McDermott MW, Parsa AT, Berger MS (2011) An extent of resection threshold for newly diagnosed glioblastomas: clinical article. J Neurosurg 115(1):3–8. 10.3171/2011.2.JNS10998 21417701 10.3171/2011.2.jns10998

[25] Schmahmann JD (2003) Vascular syndromes of the thalamus. Stroke 34(9):2264–2278. 10.1161/01.STR.0000087786.38997.9E 12933968 10.1161/01.STR.0000087786.38997.9E

[26] Serra C, Guida L, Staartjes VE, Krayenbühl N, Türe U (2019) Historical controversies about the thalamus: from etymology to function. Neurosurg Focus 47(3):E13. 10.3171/2019.6.FOCUS19331 31473672 10.3171/2019.6.FOCUS19331

[27] Serra C, Türe U, Krayenbühl N, Şengül G, Yaşargil DCH, Yaşargil MG (2017) Topographic classification of the thalamus surfaces related to microneurosurgery: a white matter fiber microdissection study. World Neurosurg 97:438–452. 10.1016/j.wneu.2016.09.101 27725299 10.1016/j.wneu.2016.09.101

[28] Serra C, Türe H, Yaltırık CK, Harput MV, Türe U (2021) Microneurosurgical removal of thalamic lesions: surgical results and considerations from a large, single-surgeon consecutive series. J Neurosurg 135(2):458–468. 10.3171/2020.6.JNS20524 33007756 10.3171/2020.6.JNS20524

[29] Serra C, Huppertz HJ, Kockro RA et al (2013) Rapid and accurate anatomical localization of implanted subdural electrodes in a virtual reality environment. Journal of Neurological Surgery Part A: Central European Neurosurgery 74(03):175–182. 10.1055/s-0032-1333124 23512592 10.1055/s-0032-1333124

[30] Yasargil MG, Adamson TE, Cravens GF, Johnson RJ, Lang A (2013) Microneurosurgery, Volume IV A: CNS Tumors: Surgical Anatomy, Neuropathology, Neuroradiology, Neurophysiology, Clinical Considerations, Operability, Treatment Options. 1. Auflage. Thieme.

[31] Carrera E, Bogousslavsky J. The thalamus and behavior.

[32] Rong J, Liu Y (2024) Advances in medical imaging techniques. BMC Methods. 1(1):10, s44330–024–00010–00017. 10.1186/s44330-024-00010-7

[33] Zanuttini L, Staartjes VE, Voglis S, Colombo E, Bardelli P, Serra C Microsurgical Endoscope-Assisted Removal of a Pulvinar Glioma Through a Supracerebellar Transtentorial Approach to the Cisternal Surface of the Thalamus: 2-Dimensional Operative Video. Oper Neurosurg. Published online May 28, 2025. 10.1227/ons.0000000000001661 10.1227/ons.0000000000001661 40434384

[34] Yasargil MG (2013) Volume IV B: CNS Tumors. 1. Auflage. Thieme.

